# The role of vascular complexity on optimal junction exponents

**DOI:** 10.1038/s41598-021-84432-1

**Published:** 2021-03-08

**Authors:** Jonathan Keelan, James P. Hague

**Affiliations:** School of Physical Science, The Open University, Milton Keynes, MK7 6AA UK

**Keywords:** Biological physics, Circulation, Statistical physics

## Abstract

We examine the role of complexity on arterial tree structures, determining globally optimal vessel arrangements using the Simulated AnneaLing Vascular Optimization algorithm, a computational method which we have previously used to reproduce features of cardiac and cerebral vasculatures. In order to progress computational methods for growing arterial networks, deeper understanding of the stability of computational arterial growth algorithms to complexity, variations in physiological parameters (such as metabolic costs for maintaining and pumping blood), and underlying assumptions regarding the value of junction exponents is needed. We determine the globally optimal structure of two-dimensional arterial trees; analysing how physiological parameters affect tree morphology and optimal bifurcation exponent. We find that considering the full complexity of arterial trees is essential for determining the fundamental properties of vasculatures. We conclude that optimisation-based arterial growth algorithms are stable against uncertainties in physiological parameters, while optimal bifurcation exponents (a key parameter for many arterial growth algorithms) are affected by the complexity of vascular networks and the boundary conditions dictated by organs.

## Introduction

**Figure 1:**
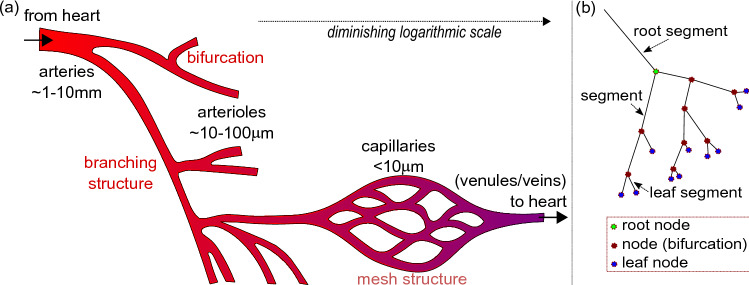
(**a**) Schematic showing the relative sizes and structures of arteries, arterioles and capillaries. The scale of the features diminishes logarithmically from left to right. Arteries supply organs and typically have sizes greater than approximately 1 mm (with the aorta the largest artery with a 2–3 cm diameter). Arterioles are typically less than 100 $\upmu$m in diameter and distribute blood within organs via a branching structure. We note that the boundary between scales of arteries and arterioles is blurred. Capillaries have a mesh like structure and control the distribution of nutrients, particularly oxygen, to tissue. After leaving the capillaries, blood enters the venous system before returning to the heart. (**b**) Schematic showing how the vessels and bifurcations of an arterial tree can be represented as segments and nodes. Segments are shown as lines and nodes as circles.

Vascular systems connect large numbers of tiny capillaries to small numbers of arteries and are therefore highly complex. This complexity and scale range is described schematically in Fig. 1. Capillaries in humans can have diameters as small as $\sim \,5\,\upmu {\mathrm {m}}$^1^, whereas the aorta is 2–3 cm wide^2^, so the radii of vessels in the vascular network of the human body cover almost four orders of magnitude^2^. Within a typical organ, vascular trees connect major arteries of $\sim \,1-10{\mathrm {mm}}$ diameter to huge numbers of tiny arterioles with width of $\sim \,10-100\,\upmu {\mathrm {m}}$ which themselves connect to capillaries of $\lesssim \,10\,\upmu {\mathrm {m}}$ (see e.g. Ref.^2^). Networks of arteries and arterioles typically have a tree like structure, which branches or bifurcates^3^. At each bifurcation vessels get proportionally smaller, and the result is that vessel size diminishes exponentially until reaching the capillaries. Capillaries have a distinct mesh like structure and are optimised for transfer of oxygen and other nutrients to tissue. There is no hard boundary between the length scales of arteries and arterioles, since the vessel structure is very similar. However broadly speaking, arteries supply blood to organs, arterioles distribute blood within organs, and capillaries distribute blood to tissues.

Vascular networks within organs are primarily constructed from bifurcations^3^, which can be characterised by defining a bifurcation exponent, $\gamma$, which is also known as the radius exponent or junction exponent (see e.g. Ref.^4^). A bifurcation consists of a single input vessel that branches into two child vessels, which we shall label A and B. The radii of the two output vessels, $r_{\mathrm{out,A}}$ and $r_{\mathrm{out,B}}$, are related to the radius of the input vessel, $r_{\mathrm{in}}$, via,

1 $$\begin{aligned} r_{\mathrm{in}}^{\gamma } = r_{\mathrm{out,A}}^{\gamma }+r_{\mathrm{out,B}}^{\gamma }. \end{aligned}$$

The goal of this paper is to carry out a theoretical and numerical analysis to determine the optimal bifurcation exponent for the efficient supply of blood through large and complex arterial trees. Evolution makes compromises between different physical and physiological costs on many length scales when optimising arterial networks. Key costs are due to pumping blood (which is a viscous fluid) and the metabolic requirements of blood manufacture^5^. The physics of pumping blood through large vessels is often dominated by pulsatile flows and turbulence, while small vessels are microfluidic^6^, which may lead to additional costs.

The simplest analysis of the competition between the cost of blood flow and metabolic costs of blood maintenance in single cylindrical vessels, predicts an optimal junction exponent of, $\gamma _{\mathrm{opt}}=3$^5^. In the classic analysis of Ref.^5^, two competing contributions to the metabolic demand of vessels are examined: the power dissipated during flow through the cylinder, and the metabolic cost of maintaining a volume of blood. The former is minimised for wide vessels and the latter for narrow vessels, so the actual radius is a compromise. This analysis leads to the conclusion that flow is proportional to the cube of vessel radius, and is known as Murray’s law. When combined with conservation of flow, Murray’s law leads to the prediction that $\gamma =3$ in Eq. (1). More detail on Murray’s law can be found in the "Power cost" and "Murray’s law" sections of this paper.

In living organisms, the bifurcation exponent associated with arteries and arterioles is often measured to deviate from three^6^, which is not fully understood, although several factors are known to lead to $\gamma _{\mathrm{opt}}\ne 3$ in single-vessel analyses. Reference^6^ reviews a range of studies that measure $\gamma$ in various organs and species, with $\gamma$ ranging from $\sim 2.1$ to $\sim 3.5$ in systemic arterial trees (with a greater range in the pulmonary vasculature). There are two key lines of argument to explain why $\gamma <3$. The effects of pulsatile flow, elastic wall vessels, and turbulence all contribute to additional power dissipation in an arterial segment, leading to a reduction in the optimal junction exponent to $\gamma _{\mathrm{opt}}=2.33$^6^. Alternatively, it has been argued that cross-sectional area is conserved at bifurcations, i.e. $\gamma _{\mathrm{opt}}=2$^7^. Curiously, in some organs, $\gamma$ is measured to be slightly greater than three^6,8^. To our knowledge, no explanation of this effect is available, since corrections to flow in single artery analyses to include turbulence, pulsatile flow, and elastic wall vessels, lead to $\gamma _{\mathrm{opt}}<3$. This suggests that single vessel analysis may be insufficient to explain $\gamma _{\mathrm{opt}}>3$ and thus that complex networks representing large numbers of arteries may be needed to properly analyse vascular trees.

We propose that, in order to fully understand the optimal branching exponents in vascular trees, it is essential to take into account the complex structure of the entire arterial network in an organ, and the boundary conditions imposed by the organism on the arteries that enter organs (particularly on the flow and artery radius). A single vessel is part of a much larger arterial tree for an organ, that is in turn part of an organism, and the role of this additional complexity on optimal supply networks is poorly understood. Two factors define boundary conditions for arterial growth algorithms: (1) The metabolic demand of the organ determines the blood flow to the organ. (2) The radius of the primary artery supplying that organ is determined by a compromise between the whole organism and the organ.

There are a huge number of possible ways to connect arterioles to arteries, yet not all are optimal. Our goal is to examine the complex multiscale structures of vascular networks that emerge from optimisation considerations both computationally and analytically. The process of optimisation in complex trees can be difficult to reproduce computationally. When optimising, the space of possible connections between the capillaries and the arteries needs to be explored. The number of possible combinations of vessels associated with these connections is enormous, and all vessels contribute to metabolic and pumping costs. It is not possible to search through every combination for all but the smallest trees. Moreover, deterministic minimisation methods (e.g. steepest descent) run the risk of finding local rather than global minima. Thus, stochastic optimisation algorithms (such as simulated annealing) are needed^9^.

Stochastic optimisation algorithms are highly advanced optimisation approaches that use random variables to quickly search through the configuration space of a problem to find (near) globally optimal solutions^10^. In particular, simulated annealing is guaranteed to approach the global optimum as computational times are increased^11^. We previously introduced the Simulated AnneaLing Vascular Optimisation (SALVO) algorithm to find the globally optimal structure of arteries using simulated annealing to overcome these problems^8,9^. The power of the numerical SALVO algorithm is that the generated trees are globally optimised and therefore represent the lowest possible total power cost associated with the whole vasculature. While the globally optimised solution represents an idealised evolutionary endpoint, insight into the compromise associated with optimising the competing costs of the different metabolic requirements associated with complicated vasculatures can be gained by this analysis. A summary of SALVO can be found in the "SALVO" section, with a detailed introduction in Ref.^8^.

Our work goes beyond previous analyses^5,6,12–15^, by optimising entire trees, rather than a single arterial bifurcation. The constraints on flow and radius of root vessels in real organs are also taken into account. Further to this analysis, we use the SALVO algorithm^8,9^ to determine the globally optimal bifurcation exponent, which allows us to check any analytical expressions that we have calculated against numerically exact values.

This paper is organised as follows: In the "Methods" section we introduce the methodology used in this paper. The "Analytical results" section presents analytical expressions for the properties of large vasculatures. The "Numerical results" section presents results from the SALVO algorithm applied to two-dimensional planes. Finally we present a "Discussion and conclusions" section.

## Methods

In this section, we discuss some of the basic formalism related to the calculation of optimal $\gamma$, and its relation to the SALVO algorithm.

### Power cost

In order to make it possible to calculate the properties of large and complex arterial trees, the arterial tree is divided into straight segments and bifurcations, such that segments join bifurcations. Thus each bifurcation becomes a node within a tree network. In the following, we shall interchangably use the terms node and bifurcation. The largest vessel from which blood arrives shall be referred to as the root node. The smallest vessels at the end of the tree shall be identified as leaf nodes. A schematic of the terminology associated with the arterial network can be found in Fig. 1b.

Poiseuille flow is assumed within each segment. It is also assumed that maintaining blood has a metabolic cost proportional to its volume. With these assumptions, the power cost for pumping blood through a single arterial tree segment is^5^,

2 $$\begin{aligned} W_{j} = m_b \pi r_{j}^2 l_{j} + \frac{8 \mu f_{j}^{2} l_{j}}{\pi r_{j}^{4}} \end{aligned}$$

where *j* denotes a segment, $r_{j}$ the segment radius, $l_{j}$ its length, $f_{j}$ its volumetric flow, $m_b$ the metabolic power cost to maintain a volume of blood, and $\mu$ the dynamic viscosity of blood. The first term in this expression represents the metabolic cost to maintain a volume of blood, and has units of energy expended per unit time (power). The second term represents the power required to pump blood through the segment. The power cost associated with bifurcations is neglected.

The total cost, ${\mathscr {W}}$, of an arterial tree is the sum of these individual segment costs,

3 $$\begin{aligned} {\mathscr {W}}= \sum _{j \in \{{\mathrm{segments}}\} } W_{j}. \end{aligned}$$

Equation 3 will be referred to as the cost function.

### Murray’s law

Murray’s law ($f\propto r^3$) is derived by optimising total power expenditure for pumping blood through a single segment, as given by Eq. (2)^5^. By differentiating Eq. (2) with respect to $r_{j}$,

4 $$\begin{aligned} \frac{\partial W_{j}}{\partial r_{j}}=2m_{b}\pi f_{j}^{2}l_{j}-\frac{32\mu f_{j}^2l_{j}}{\pi r_{j}^{5}}. \end{aligned}$$

When $\partial W_{j}/\partial r_{j}=0$, the optimal relation between $f_{j}$ and $r_{j}$ can be found. This leads to Murray’s law,

5 $$\begin{aligned} f_{j}=\frac{m_{b}^{1/2}\pi }{4\mu ^{1/2}}r_{j}^{3} \end{aligned}$$

In the following analysis, we will assume that $l=l_{\mathrm{root}}r^{\alpha }/r_{\mathrm{root}}^{\alpha }$, where $l_{\mathrm{root}}$ and $r_{\mathrm{root}}$ are the length and radius of the root segment respectively, and $\alpha$ is the length–radius exponent^6^. This slightly modifies the preceding argument, so that,

6 $$\begin{aligned} f_{j}=\frac{m_{b}^{1/2}\pi }{2(2\mu )^{1/2}}r_{j}^{3}\sqrt{\frac{2+\alpha }{4-\alpha }} = \frac{f_{\mathrm{root}}}{r_{\mathrm{root}}^{3}}{r_{j}^{3}}, \end{aligned}$$

where $f_{\mathrm{root}}$ is the flow through the root segment.

### SALVO

The (SALVO) algorithm developed in earlier papers for three dimensional arterial trees^8,9^ can also be used to generate arterial trees in two-dimensional planes. In this section, an outline of this algorithm is given. The algorithm is similar to the approach for growing cardiac and cerebral vasculature^8,9^, with some differences relating to the use of fixed nodes to supply tissue. A detailed description of the SALVO algorithm can be found in Ref.^8^.

**Figure 2:**
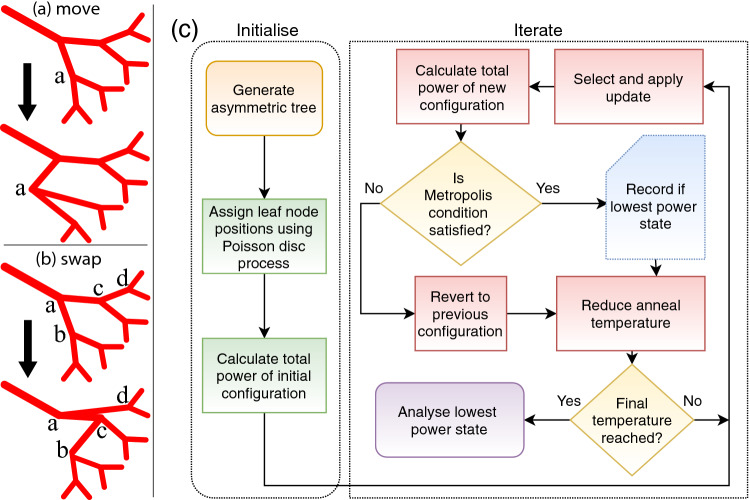
Schematic of the algorithm, showing the two types of update and a flow diagram of the SALVO process. (**a**) and (**b**): Two types of update are required for ergodicity, that (**a**) move node coordinates and (**b**) swap the parent segments of nodes. The figure shows a summary of these updates. In panel (**a**) node a is moved. In panel (**b**) parents of two nodes (nodes b and d) are swapped. The parent of node b is node a, and the parent of node d is node c. After the swap, the parent of node b is node c and the parent of node d is node a. (**c**) Flow diagram showing the initialisation and iterative processes associated with SALVO.

**Table 1:** Simulation parameters and their ranges.

Name	Symbol	Range
Bifurcation exponent	$\gamma$	1.0–5.0
Metabolic ratio	$\Omega$	0.1–10
Number of leaf nodes	*N*	100–5000
Blood viscosity	$\mu$	$3.6\times 10^{-3}$ Pa s
Tissue size	*a*	1 cm
SA steps	$\nu$	$10^{8}$ ($10^{9}$ for checks)
SA initial ‘temperature’	$T_{0}$	1 Js$^{-1}$
SA final ‘temperature’	$T_{\nu }$	$10^{-12}$ Js$^{-1}$
Short move distance	$d_{\mathrm {move}}$	0.05 mm
Long move distance	$d_{\mathrm {move}}$	0.5 mm
Short move node weight		0.3
Long move update weight		0.2
Swap update weight		0.5

In a key difference to previous work, we study an idealised two-dimensional (2D) piece of ‘tissue’, with fixed leaf-node positions. The root node of the tree is fixed to the corner of a square region of side $a(=10\,{\text {mm}})$. A Poisson disc process is used to place leaf nodes^16^. The whole 2D region is accessible by nodes, with only metabolic- and flow-related penalties in the cost function, Eq. (3). Since the leaf nodes are evenly distributed by the Poisson disc process, and do not move during the update process, a penalty for under- and over-supply (see Ref.^9^) is not required. Also, no penalties are required to stop penetration of large vessels into the tissue (as in Ref.^8^) or cavities within the tissue (such as the ventricles of the heart in Ref.^9^) since all vessels lie within a continuous 2D tissue. Overall this decreases the complexity of the algorithm.

On each iteration, modifications to the binary tree are attempted by either (1) selecting a node at random and then moving it or (2) selecting two nodes at random and then changing the tree structure by swapping the parents of those nodes. These updates are sufficient to ensure ergodicity. Updates are summarised in Fig. 2 and relevant parameters are summarised in Table 1. The root node is never updated. An example of moving a node is shown in Fig. 2a. In the update, the initial configuration at the top of the figure changes to the final configuration at the bottom of the figure, by moving the location of the node labelled a. The distance moved is short (0.05 mm) in 30% of updates, and long (0.5 mm) in 20% of updates. In the version of the algorithm used in this paper, leaf nodes are never moved. An example of swapping the parents of a node is shown in Fig. 2b. In this case, it is the parents of nodes labelled b and d that are swapped. In the initial configuration, node c is the parent of node d, and node a is the parent of node b. After the update, node a is the parent of node d and node c is the parent of node b. The swapping of parent nodes is attempted on $50\%$ of iterations.

We use simulated annealing to optimise the cost function^17^. Within this framework, acceptance of the updates is determined according to the Metropolis condition,

7 $$\begin{aligned} P_{\theta ,\theta +1} = \mathrm{min}\left\{ \exp (\frac{-\Delta {\mathscr {W}}^{(\theta ,\theta +1})}{T_{\theta }}),1\right\} \end{aligned}$$

where $\Delta {\mathscr {W}}^{(\theta ,\theta +1)}={\mathscr {W}}^{(\theta +1)}-{\mathscr {W}}^{(\theta )}$ is the change in cost associated with modifying the tree from the configuration in iteration $(\theta )$ to the configuration proposed for iteration $(\theta +1)$, and ${\mathscr {W}}$ as defined in Equation 3 is the cost function at the core of the SALVO algorithm. In practice, a uniform random variate $r\in [0,1)$ is calculated and if $P>r$ the proposed configuration is accepted. If the proposed configuration is rejected, then the configuration of iteration $\theta$ is carried forward to iteration $\theta +1$. $T_{\theta }$ is the annealing temperature, which is slowly reduced using the common exponential schedule, $T_{\theta + 1} = \epsilon T_{\theta }$ where $\theta$ is the iteration number, $\epsilon = \exp ( \ln T_{0} - \ln T_{\nu })/\nu$, $\nu$ the total number of iterations, and $T_{0}$ ($T_{\nu }$) are the initial (final) temperatures. Updates are iteratively applied until the final temperature is reached. The algorithm is summarised in the flow diagram of Fig. 2c.

As we have discussed elsewhere^8^, limitation of the method to a few thousand nodes is related to the combinatorial (factorial) growth of the number of possible tree configurations. 5000 nodes trees are already quite detailed, and determination of $\gamma _{\mathrm{opt}}$ for such trees already offers a substantial computational challenge: The required $10^8$ updates take approximately 11 hours on a single thread of a Threadripper 2990WX processor, and we have to distribute the optimisation of large numbers of similar trees with different $\gamma$ across the full 64 threads of the processor to find a single value of $\gamma _{\mathrm{opt}}$ (and are then calculating for many different physiological parameters). To make these calculations with much larger numbers of nodes, very significant advances in computational power would be needed. The primary issue is that the number of updates needed to optimise the tree grows with the number of possible combinations. Therefore, the optimisation of larger trees is not just a matter of greater computational power, since the required computational power grows so rapidly. A possibility is application of alternative optimisation algorithms, e.g. genetic algorithms, ant-trail optimisation etc, although these are much harder to implement for this problem. Unfortunately, even if certain optimisation algorithms are faster, all will eventually be limited by the combinatorial growth of possible tree configurations. In the context of this paper, we need to identify the global minimum, so approximate strategies such as multiscale algorithms^18^ or constrained constructive optimisation (CCO)^19^ are not appropriate.

## Analytical results

In this section, we discuss analytic approximations to the total power of large and complex vascular trees. This provides initial insight into the deviations in $\gamma _{\mathrm{opt}}$ caused by tree complexity. We start by discussing a formalism for simplifying the total power calculation of large and complex arterial trees.

### Formalism and simplifications

Arteries can be grouped together, so that each group comprises arteries with identical properties (e.g length, diameter, flow). In a real arterial system, this would not be exact, but it would still be possible to group arteries with similar lengths, radii, and flows together. By using this grouping, the total power can be rewritten as,

8 $$\begin{aligned} {\mathscr {W}}= \sum _{j \in \{r,l,q\}} {\mathscr {N}}(r_j,l_j,f_j) \left( m_b \pi r_{j}^{2} l_j + \frac{8 \mu f_{j}^{2} l_j}{\pi r_{j}^{4}} \right) , \end{aligned}$$

where ${\mathscr {N}}(r_j,l_j,f_j)$ is the number of arterial segments with identical radii, lengths and flows.

Under the restriction that the flow in all leaf nodes is identical and equal to $f_{\mathrm{leaf}}$, the flow in each segment is,

9 $$\begin{aligned} f_{n} = n f_{\mathrm{leaf}} \end{aligned}$$

where *n* is an integer and represents the total number of leaf nodes downstream of the segment.

Comparing Eq. (1) with flow conservation, a radius–flow relation is identified:

10 $$\begin{aligned} f_{n} = f_{\mathrm{leaf}} (r_{n}/r_{\mathrm{leaf}})^{\gamma }. \end{aligned}$$

Thus, by substituting Eq. (9) into Eq. (10), the radius can be rewritten in terms of *n* and $\gamma$:

11 $$\begin{aligned} r_{n}=r_{\mathrm{leaf}}\left( f_{n}/f_{\mathrm{leaf}}\right) ^{1/\gamma } = r_{\mathrm{leaf}}n^{1/\gamma }. \end{aligned}$$

Experimental data suggest that the length of an arterial segment is proportional to a power of the radius,

12 $$\begin{aligned} l_{n} = l_{\mathrm{leaf}} \left( r_{n}/r_{\mathrm{leaf}}\right) ^{\alpha } = l_{\mathrm{leaf}} \left( f_{n}/f_{\mathrm{leaf}}\right) ^{\alpha /\gamma }, \end{aligned}$$

where the value of the exponent $\alpha$ is typically close to 1.0^6,20^.

By substituting Eqs. (11) and (12) into Eq. (2), the power required to maintain blood flow through a segment is found to depend only on the flow $f_{n}$,

13 $$\begin{aligned} W_{n} = W(f_{n}) = m_{b}\pi r_{\mathrm{leaf}}^{2}l_{\mathrm{leaf}} \left( f_{n}/f_{\mathrm{leaf}}\right) ^{(2+\alpha )/\gamma } +\frac{8\mu l_{\mathrm{leaf}}}{\pi r_{\mathrm{leaf}}^{4}} f_{n}^{2} \left( f_{n}/f_{\mathrm{leaf}}\right) ^{(\alpha -4)/\gamma }. \end{aligned}$$

Thus, the dimensionless *metabolic ratio*, defined as $\Omega = m_{b}\pi ^2 r_{\mathrm{leaf}}^{6}/8\mu f_{\mathrm{leaf}}^2$, the *bifurcation exponent*, and the number of nodes, *N*, define the parameter space. The power associated with a segment is then,

14 $$\begin{aligned} W_{n} & = C \left( \Omega n^{(2+\alpha )/\gamma }+n^{2+(\alpha -4)/\gamma }\right) \end{aligned}$$

15 $$\begin{aligned} & = C n^{1+(\alpha -1)/\gamma }\left( \Omega n^{3/\gamma -1}+n^{1-3/\gamma }\right) \end{aligned}$$

where $C=8 \mu f_{\mathrm{leaf}}^2 l_{\mathrm{leaf}}/\pi r_{\mathrm{leaf}}^4$. Both *C* and $\Omega$ are defined in terms of the leaf node properties. A similar ratio for the root node, $\Omega _{\mathrm{root}}=m_{b}\pi ^2 r_{\mathrm{root}}^{6}/8\mu f_{\mathrm{root}}^2$ can be defined for convenient contact with experiment. The values $r_{\mathrm{root}}$ and $f_{\mathrm{root}}$ are often known from experiment, e.g. Doppler ultrasound, and *N* can be estimated. This ratio can be related to $\Omega$ via $\Omega _{\mathrm{root}}=N^{6/\gamma -2} \Omega$.

The total power required to supply the whole vascular tree is,

16 $$\begin{aligned} {\mathscr {W}}= \sum _{n} {\mathscr {N}}_n W_{n}. \end{aligned}$$

${\mathscr {N}}_{n}$ is the number of segments with flow $n f_{\mathrm{leaf}}$, and simplifies the function ${\mathscr {N}}(r_i,l_i,f_i)$. For any tree structure, *N* is always the number of leaf nodes, so ${\mathscr {N}}_{1}=N$. There is always a single root node with total flow $N f_{\mathrm{leaf}}$, so ${\mathscr {N}}_{N} = 1$. No node has flow greater than $N f_{\mathrm{leaf}}$, so ${\mathscr {N}}_{n>N}=0$. The remaining ${\mathscr {N}}_n$ are dependent on the structure of the tree. At each bifurcation, flow conservation requires that $n_{\mathrm{in}}=n_{\mathrm{out,1}} + n_{\mathrm{out,2}}$, so *n* is an integer.

Total power is linear in length scale, so the location of any minima in the power with respect to $\gamma$ is independent of *a*. It is the global minimum with respect to $\gamma$ that sets the structure of the tree, and when locating the minimum, $\partial {\mathscr {W}}/\partial \gamma =0$, so the factor of $l_{\mathrm{leaf}}$ in *C* simply cancels, thus making the solution independent of *a*. Changes in $r_{\mathrm{leaf}}$ can be absorbed into the ratio $m_b/\mu$ and thus are similar to changing the metabolic requirements of the organ^9^.

There are two special tree structures: the fully symmetric and fully asymmetric trees. In the first case, identified as a fully symmetric tree, the flow is split evenly at each bifurcation. For the case which we shall identify as fully asymmetric, a single leaf node emerges at each bifurcation and the rest of the flow passes down the other bifurcation. We will explore these special cases in the following two sections.

### Fully symmetric vascular tree

In a fully symmetric tree, all of the segments with flow *n* exist at the same bifurcation layer. Each layer, denoted by the integer *m*, has $2^{m}$ segments, where *m* is the number of bifurcations upstream of that layer ($m = 0$ at the root segment). Within a layer, all segments have the same flow, and thus the same radius and length. The tree has a total of *M* layers, so $0\le m\le M$. Therefore ${\mathscr {N}}_{n} =2^{m}$ if $n=2^{M-m}$, ${\mathscr {N}}_{n}=0$ otherwise, and the total power cost in Eq. (16) becomes,

17 $$\begin{aligned} {\mathscr {W}}= C\sum _{m = 0}^{M} 2^{m} \left( \Omega 2^{(M-m)(2+\alpha )/\gamma }+2^{(M-m)(2+(\alpha -4)/\gamma )} \right) . \end{aligned}$$

By summing this geometric series, the total power cost for a fully symmetric tree is found to be,

18 $$\begin{aligned} {\mathscr {W}}= 2^{M} C\left( \Omega \frac{2^{-M(1-(2+\alpha )/\gamma )}-1}{1-2^{1-(2+\alpha )/\gamma }}+ \frac{2^{M(1+(\alpha -4)/\gamma )}-1}{1-2^{-(1+(\alpha -4)/\gamma )}}\right) \end{aligned}$$

### Fully asymmetric tree

The total power cost of the fully asymmetric tree may be calculated by noting that each discrete flow is represented once for all *n*, so ${\mathscr {N}}_{n} = 1$, for $n<N$. The exception being that there are *N* leaf nodes so ${\mathscr {N}}_{1}=N$.

Substitution into Eq. (16) gives,

19 $$\begin{aligned} {\mathscr {W}}= C\left( \sum _{n=1}^{N} (\Omega n^{(2+\alpha )/\gamma }+n^{2+(\alpha -4)/\gamma }) + (\Omega +1)(N-1)\right) \end{aligned}$$

So the total power cost for an asymmetric tree is

20 $$\begin{aligned} {\mathscr {W}}= C\left( \Omega H_{N}^{(-(2+\alpha )/\gamma )}+H_{N}^{(-(2+(\alpha -4)/\gamma ))}\right) + C(\Omega +1)(N-1), \end{aligned}$$

where $H_{n}^{(r)}$ is the generalized harmonic function, $\sum _{k=1}^{n} 1/k^{r}$.

### Optimal bifurcation exponent

**Figure 3:**
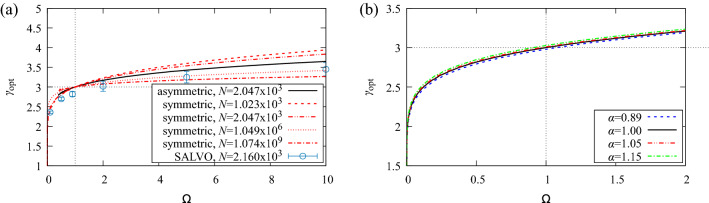
(**a**) Deviations from Murray’s law ($\gamma _{\mathrm{opt}}=3$) depend strongly on the metabolic ratio, $\Omega$, but are essentially independent of the structure of the tree. The figure shows a comparison of $\gamma _{\mathrm{opt}}$ vs $\Omega$ for fully symmetric, asymmetric and numerical trees. (**b**) Optimal bifurcation exponent does not depend strongly on $\alpha$.

The optimal value of $\gamma$ is obtained by numerically solving $\partial {\mathscr {W}}/\partial \gamma =0$ for Eqs. (18) and (20) . Results are shown in Fig. 3 for symmetric and asymmetric trees, for various $\Omega , \alpha$ and *N*.

**Figure 4:**
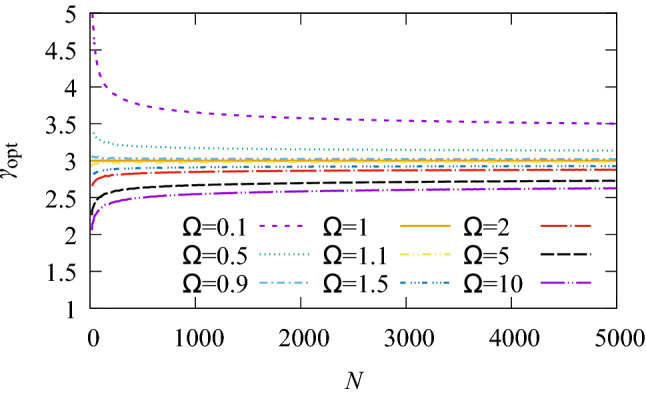
Deviations from Murray’s law are largest for small trees and strongly dependent on changes in the metabolic ratio. The figure shows $\gamma _{\mathrm{opt}}$ vs *N* for a fully asymmetric tree.

The optimal bifurcation exponent is strongly dependent on the metabolic ratio, $\Omega$, which can change due to physiological boundary conditions on flow and radius at the input vessels. These constraints may be due to limits in the size of the largest vessel in the tree imposed by the physiology of the whole organism and the flow demands of the organ. Figure 3a shows the optimal value of $\gamma$. When $\Omega =1$ and $\alpha =1$ the result of Murray’s law ($\gamma _{\mathrm{opt}}=3$) is recovered.

$\gamma _{\mathrm{opt}}$ is qualitatively unchanged by the structure of the tree. Results for asymmetric and symmetric trees with $N=2.047\times 10^{3}$ follow essentially the same functional forms. The optimal bifurcation exponent for the asymmetric tree is closer to $\gamma =3$ than the symmetric tree. Also shown in Fig. 3a are numerical values from SALVO, which will be discussed later.

The optimal bifurcation exponent is modified away from $\gamma =3$ by changes in the length exponent, $\alpha$ (Fig. 3b). This structural effect potentially has implications for the value of $\gamma _{\mathrm{opt}}$ in organs, since $\alpha$ can vary with organ type, with estimates ranging from 0.89–1.15. In practice, changes in $\gamma _{\mathrm{opt}}$ for this variation in $\alpha$ are far smaller than the error for measurements of $\gamma$ and changes in $\alpha$ can essentially be neglected.

Deviations from Murray’s law are largest for small trees and strongly dependent on changes in the metabolic ratio. The larger the tree, the closer to Murray’s law $\gamma _{\mathrm{opt}}$ becomes. Fig. 4 shows variation of $\gamma _{\mathrm{opt}}$ with *N* for fully symmetric trees. For vascular tree sizes of between $10^{3}$ and $10^{6}$ segments, which are typical in organs, $\gamma _{\mathrm{opt}}$ ranges between 2 and 4.

## Numerical results

The generation of globally optimal trees using a numerical algorithm helps to test analytic expressions, and provides additional morphological measures that can be used to understand arterial networks. In this section, we use SALVO to investigate the role of vascular complexity and physiological boundary conditions on the properties of globally optimal trees. Several properties of the numerically generated trees are investigated. We determine the dependence of globally optimal tree structures on $\Omega$ and $\gamma$. Through examination of ${\mathscr {W}}$, we compute $\gamma _{\mathrm{opt}}$ for complex trees. For each value of $\gamma$ and $\Omega$ investigated, arterial trees with up to 5000 nodes were generated. Table 1 summarises the parameters used for the numerical calculations. We note that we carried out checks on convergence with a subset of trees by using an anneal schedule with a larger number of steps ($\nu =10^{9}$), finding no major changes to the tree structure.

### Tree morphology

**Figure 5:**
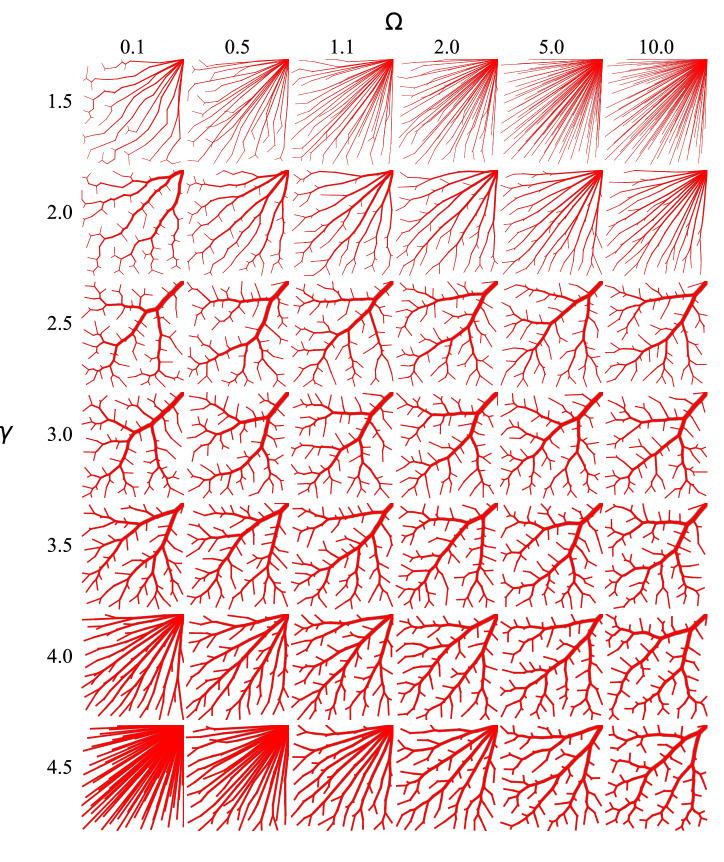
The structure of the globally optimal vasculature varies with $\gamma$ and $\Omega$. Trees have size $N=100$. Radii are normalised by the root radius for easier visualisation.

There are three regions of the parameter space with qualitatively different tree structures, examples of which can be seen in Fig. 5. In the figure, the vessel widths are normalised to the root radius to improve visibility. Trees are generated for $N=100$ and various $\Omega$ and $\gamma$:

- **Star regions** ($\gamma \lesssim 2, \Omega >1$ and $\gamma \gtrsim 4, \Omega <1$): long and narrow leaf segments originate from the vicinity of the root node.
- **Asymmetric regions** ($\gamma \lesssim 2, \Omega <1$ and $\gamma \gtrsim 4, \Omega >1$): asymmetric and tortuous branches dominate.
- **Physiological region** ($2\lesssim \gamma \lesssim 4$): trees have a branching structure similar to the kinds of vasculature seen in living tissue.

In the star regions ($\gamma \lesssim 2, \Omega \gg 1$ and $\gamma \gtrsim 4, \Omega \ll 1$), long leaf segments connect root and leaf nodes (see top right panels in Fig. 5). This is due to the domination of the $n^{3/\gamma -1}$ term (that represents metabolic maintenance of blood) for low $\gamma$, and the $n^{3/\gamma '-1}$ Poiseuille term for large $\gamma$. Thus, terms with small *n* (i.e. leaf nodes) are favoured. Trees in both star regions are very similar, which is not a coincidence, and can be explained by examining the structure of Eq. (15). When $\alpha \approx 1$, the power in a segment is $W_{n} = C n(\Omega n^{3/\gamma -1}+n^{1-3/\gamma })$. For $\gamma >3$, the exponents (which involve $3/\gamma -1$) have opposite sign to those for $\gamma <3$. So after the substitutions $\Omega =1/\Omega '$, $\gamma =3\gamma '/(2\gamma '-3), C'=\Omega C$, $W_{n} = C' n(\Omega ' n^{3/\gamma '-1}+n^{1-3/\gamma '})$, and the sum has an equivalent structure. The substitution is determined by identifying where $1-3/\gamma =3/\gamma '-1$. Since the prefactor $C'$ scales the entire sum, then the minima of ${\mathscr {W}}$ and thus the results for $\gamma ,\Omega$ and $\gamma ',\Omega '$ are identical. This symmetry is only approximate if $\alpha \ne 1$.

Trees in the asymmetric regions ($\gamma \lesssim 2,\Omega \ll 1$ and $\gamma \gtrsim 4,\Omega \gg 1$), have a highly asymmetric structure, with long trunks snaking through leaf node sites (see top left panels in Fig. 5). This is due to the domination of the $n^{1-3/\gamma }$ term due to Poiseuille flow for low $\gamma$, and the $n^{1-3/\gamma '}$ metabolic cost term for large $\gamma$. Thus terms with large *n* (i.e. thick trunks) are favoured. A similar argument to that given for the star regions explains why the trees in both asymmetric regions have very similar structures.

In the physiologically relevent region ($2<\gamma <4$), trees have a more symmetric structure. No single term in ${\mathscr {W}}$ dominates. There is surprisingly little variation between the tree structures for different $\gamma$ and $\Omega$ within this region. The vascular structures are reminiscent of those in the retina^21^, although we postpone analysis of the vasculature of the curved retina for future work.

**Figure 6:**
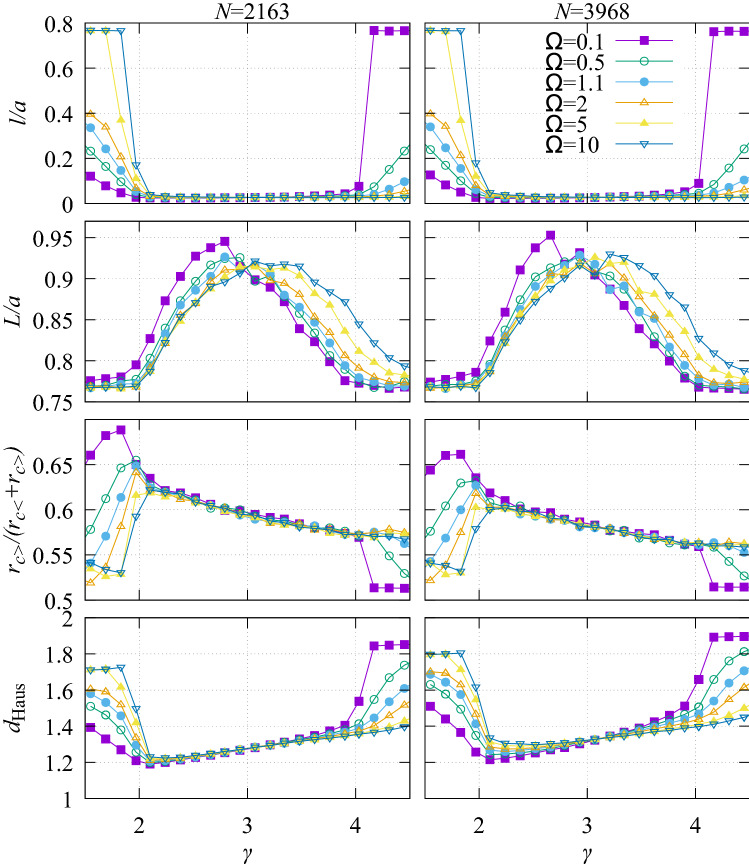
The tree morphology is dependent on the bifurcation exponent, but essentially independent of variation in $\Omega$ within the region of physiological interest between $\gamma =2$ and $\gamma =4$. Morphology is strongly dependent on $\Omega$ outside this range. There is only minor dependence on tree size at any values of $\gamma$ and $\Omega$.

To quantify the effect of varying $\gamma$ and $\Omega$ on the network structure, we have examined average segment length, path length, radius asymmetry and Hausdorff dimension (Fig. 6). Average length is defined as $l=\sum l_j/N$. The average summed path length from root to leaf node is $L=\langle \sum _{\mathrm {path}} l_j \rangle$. Radius asymmetry is measured using $\langle r_{c>}/(r_{c<}+r_{c>})\rangle$ (where at each bifurcation the larger and smaller of the radii of child vessels are labelled $r_{c>}$ and $r_{c<}$, such that $r_{c>} \ge r_{c<}$). The Hausdorff dimension is calculated using a box counting method.

In the physiological region, the dominant factor controlling morphological properties is $\gamma$. Within that range, morphological properties do not depend strongly on $\Omega$. Average segment length is short and path length is long in this region, consistent with the branching structures seen for intermediate $\gamma$ in Fig. 5. Bifurcation symmetry is in the range 0.58–0.62, so bifurcations are moderately symmetric. Although $\Omega$ leads to minor changes in tree morphology in this regime, we note it can affect $\gamma _{\mathrm{opt}}$ and thus the tree morphology via $\gamma$ as a secondary effect.

In the star and asymmetric regions, $\Omega$ is responsible for large variations in the tree morphology, and $\gamma$ can also produce large variations in the various morphological and structural properties of the tree. Path length drops outside this region to approximately $a/\sqrt{2}$ consistent with a large number of straight paths from the root node to leaf nodes. The radius asymmetry is increased in the asymmetric region relative to the physiological region, and is decreased in the star region. For all other regions of the parameter space, the asymmetry drops.

Morphological measurements are not strongly dependent on changes in *N*, consistent with additional segments adding more detail to the tree, but not qualitatively changing the tree structure. Panels on the left of Fig. 6 show results for $N=2163$ and panels to the right for $N=3968$.

The Hausdorff dimension, $d_{\mathrm Haus}$, is also calculated (lowest panels of Fig. 6). This sits in the range between $d=1$ and $d=2$. The dimension of the trees with the physiological range $2<\gamma <4$ are lower than the spidery trees found outside this range.

**Figure 7:**
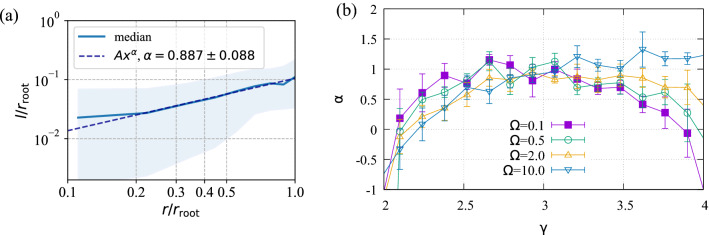
(**a**) A power law relationship is found for the median segment length in terms of segment radius calculated using SALVO. The figure shows median values of $l/r_{\mathrm{root}}$ vs $r/r_{\mathrm{root}}$, a power law fit (dashed line), and the 25th and 75th percentiles (light blue shading). To calculate the length–radius relation, segments are binned from trees with $N>2000$, $2.75<\gamma <3.25$, $\Omega =0.9$. (**b**) The length–radius exponent, $\alpha$, is close to one for trees grown with $2.5<\gamma <3.5$. To calculate the length–radius relation, segments are binned from trees with $N>2000$ and specific $\gamma$ and $\Omega$ values and fits similar to Panel (**a**) are made. Error bars represent uncertainty in the fit.

A power law, $l=Ar^{\alpha }$, is found to relate the median segment length calculated using SALVO to the segment radius. Figure 7a shows data gathered from trees with $5000>N>2000$, $2.75<\gamma <3.25$, $\Omega =0.9$. Since there are many segments in the tree, and many trees in the analysis (to improve statistics), there is a distribution of segment lengths corresponding to each radius, just as found in experiments^6^. To indicate the spread of this distribution, we shade the range of lengths that sit between the 25th and 75th percentiles of the length distribution in light blue. To determine the parameter, $\alpha$, we fit $l=Ar^{\alpha }$ to the median value, finding the exponent to be $\alpha =0.887\pm 0.088$, consistent with experimental values^6^.

The length–radius exponent, $\alpha$, is consistent with experimental values for trees grown with $2.5<\gamma <3.5$, but can become effectively negative when long leaf segments start to dominate outside this region (Fig. 7b). To calculate the length–radius relation, segments are binned from trees with $N>2000$ and specific $\gamma$ and $\Omega$ values before fitting a power law. Where exponents are negative, the relation only poorly follows a power law, and errors on $\alpha$ are large. The power law relation is well followed within the region $2.5<\gamma <3.5$, and this leads to smaller error bars. Overall, errors on $\alpha$ determined from fitting the power law are relatively large.

### Optimal bifurcation exponent

**Figure 8:**
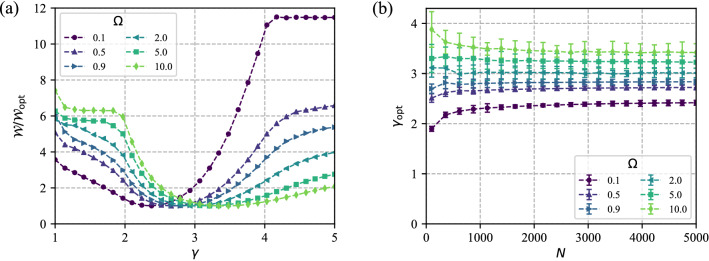
(**a**) A well defined global minimum in total power cost means that the optimal bifurcation exponent $\gamma _{\mathrm{opt}}$ can be determined without ambiguity. The figure shows the total power cost as a function of bifurcation exponent, $\gamma$, for several values of $\Omega$. (**b**) The relationship of $\gamma _{\mathrm{opt}}$ to *N* and $\Omega$, numerically determined using SALVO, is qualitatively similar to the relationship determined from analytic expressions. The figure shows $\gamma _{\mathrm{opt}}$ vs *N* for several $\Omega$. Error bars represent uncertainty in the fit to the bottom of the minimum.

The optimal bifurcation exponent $\gamma _{\mathrm{opt}}$ can be determined without ambiguity from the minimum in ${\mathscr {W}}$. Figure 8a shows how the total power cost varies with $\gamma$. There is a clearly defined global minimum for all values of $\Omega$ shown. $\gamma _{\mathrm{opt}}$ can be found by fitting a quadratic form to the bottom of the minimum.

The variation of $\gamma _{\mathrm{opt}}$ with $\Omega$ and *N*, numerically determined using SALVO, is qualitatively similar to the results from analytic expressions. Numerical values of $\gamma _{\mathrm{opt}}$ for various values of $\Omega$ vs *N* are shown in Fig. 8b, and compare favourably to Fig. 4. Several numerical values are compared with the analytic results in Fig. 3a, also showing good agreement for both symmetric and asymmetric trees.

We note that the location of the minimum in the cost function is very stable to changes in the random number seed, which determines the random number sequence used both in the Poisson disc process that initialises the leaf nodes, and as part of the Monte Carlo algorithm at the heart of simulated annealing. In practice a different seed was used for each point in Fig. 8a with no discernible fluctuation in the curves.

### Sensitivity to uncertainty in physiological parameters

#### Uncertainty in physiological parameters

There are two free physiological parameters that act as input to SALVO, $\Omega$ and $\gamma$. In this section we discuss the uncertainty in these values.

Large uncertainties on the value of $\Omega = m_{b}\pi ^2 r_{\mathrm{leaf}}^{6}/8\mu f_{\mathrm{leaf}}^2$, dominate errors in $\gamma$. Experimentally measured values for $\gamma$ in systemic arterial trees (collated in Ref.^6^) range from $\gamma \sim 2.1$ to $\sim 3.4$. Fractional errors on these values are typically of order 15% for vessels of size $>1$mm and (significantly) less than 7% for vessels of size $<1$mm. The uncertainty in $\Omega$ is itself dominated by uncertainty in the value of $m_{b}$. Estimates of the parameter $m_{b}$ can vary by up to a factor $\sim 2$^22^ (corresponding to $\sim 40\%$ fractional error). We expect radius and flow measurements to be significantly more reliable than the $m_{b}$ estimate. Thus we estimate $\Omega$ to also vary by a factor 2 due to experimental uncertainty (or $\Delta \Omega /\Omega \sim \sqrt{2}-1 \sim 0.41$).

#### Uncertainty in tree morphology

Morphological properties depend on only two parameters, $\Omega$ and $\gamma$. The uncertainties in morphological parameters can be related to variations in $\gamma$ and $\Omega$ in the usual way as, $\Delta O/O\sim \sqrt{|dO/d\gamma |^2(\Delta \gamma /\gamma )^2+|dO/d\Omega |^2(\Delta \Omega /\Omega )^2}$, where *O* represents one of the four calculated morphological properties. However, since the fractional uncertainties in measured $\gamma$ values are much smaller than those in $\Omega$, we calculate the sensitivity based on

21 $$\begin{aligned} \frac{\Delta O}{O}\sim \left| \frac{dO}{d\Omega }\right| \frac{\Delta \Omega }{\Omega }. \end{aligned}$$

**Figure 9:**
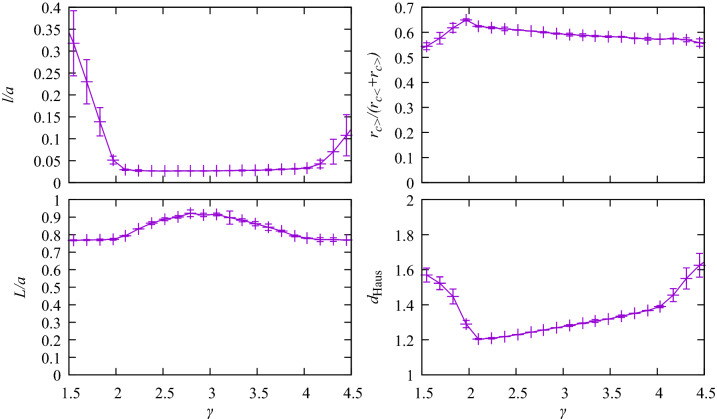
Sensitivity of morphological properties to uncertainty in $\Omega$, for $\Omega \sim 1$. Morphological properties show little sensitivity in the physiological range $2<\gamma <4$.

Figure 9 shows the sensitivity of morphological properties to uncertainty in $\Omega$, for $\Omega \sim 1$. To estimate $|d O/d\Omega |$ we used the difference between morphological measures using values of $\Omega =0.9$ and $\Omega =1.1$ to estimate $\Delta O/\Delta \Omega$, and display the average of these two measures in the figure with error bars representing the uncertainty in *O* due to $\Omega$. None of the morphological properties are very sensitive to the uncertainty in $\Omega$. There is little uncertainty within the physiological range $2<\gamma <4$. The largest uncertainties are found outside that range, but they do not lead to the possibility of qualitative changes to conclusions.

#### Uncertainty in optimal bifurcation exponent

The key result of this paper is the optimal bifurcation exponent. This is only dependent on $\Omega$, so we estimate that the uncertainty of $\gamma _{\mathrm{opt}}$ is,

22 $$\begin{aligned} \frac{\Delta \gamma _{\mathrm{opt}}}{\gamma _{\mathrm{opt}}}\sim \left| \frac{d\gamma _{\mathrm{opt}}}{d\Omega }\right| \frac{\Delta \Omega }{\Omega }. \end{aligned}$$

**Figure 10:**
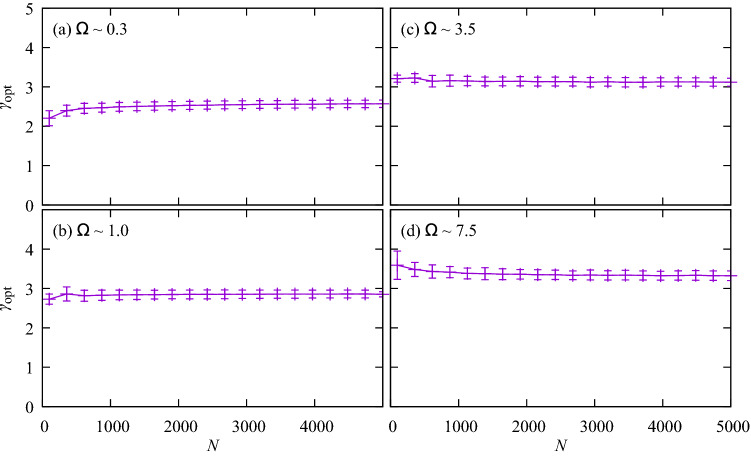
Sensitivity of $\gamma _{\mathrm{opt}}$ to uncertainty in $\Omega$ is found to be less than $10\%$ for various $\Omega$ and *N*.

We estimate uncertainties of $\gamma _{\mathrm{opt}}$ due to uncertainties in $\Omega$ to be $\lesssim 10\%$. Figure 10 shows the sensitivity of $\gamma _{\mathrm{opt}}$ to the uncertainty in $\Omega$. For tree sizes of $N=5000$, uncertainty in $\gamma _{\mathrm{opt}}$ is approximately 6%, rising to $\sim \,10\%$ for the smallest trees of $\sim \,100$ segments. Thus the results are robust against the difficulties of estimating metabolic constants.

## Discussion and conclusions

In this paper we determined analytic expressions, and carried out numerical calculations, for the properties and structures of globally optimal vascular trees, with the aim of understanding how overall complexity and physiological boundary conditions contribute to the optimal junction exponent and other properties of arterial trees. Analytic expressions were derived for the special cases of maximally symmetric and asymmetric arterial trees. The parameter space of the arterial trees was explored further by making numerical calculations with SALVO, enabling globally optimal vasculatures to be found for arbitrary tree morphology. The dependencies of tree structures, morphological properties, and optimal bifurcation (junction) exponent on physiological parameters are calculated.

The analytic expressions derived here are consistent with numerical calculations, and predict that $\gamma _{\mathrm{opt}}$ does not vary strongly with tree symmetry, so we propose that the analytic expressions derived here are applicable to a wide range of vasculatures. Analytic expressions can be used for much larger trees than numerical optimisations, and would, therefore, be useful for predicting the properties of vasculatures within a range of organs where the number of vessel segments and overall complexity exceed the capabilities of current computers. We expect that it will be possible to extend the analytic expressions to include pulsatile flow and turbulence, and will investigate this possibility in future studies.

We predict that tree complexity is a significant contributor to the bifurcation exponents measured in living organisms. The deviations originating from tree complexity are of similar size to those predicted by including turbulence and pulsatile flow in previous analyses. These deviations are particularly significant if physiological boundary conditions lead to $\Omega \ne 1$. This may occur since all organs, with their dramatically varying demands, are connected to the same major vasculature: so flow and radius associated with the arteries supplying organs may reflect compromise within an organism. We expect that large variations of $\gamma _{\mathrm{opt}}$ with increasing complexity will also occur if a more detailed analysis including pulsatile flow and turbulence is carried out.

We predict that arterial tree complexity can lead to optimal bifurcation exponents, $\gamma _{\mathrm{opt}} > 3$, a situation which can be found in experiment, and is of interest since inclusion of turbulence and pulsatile flow in single artery analyses leads to $\gamma _{\mathrm{opt}}<3$. Large values of $\gamma$ are measured in e.g. the brain vasculature ($\gamma = 3.2$)^8^, retina ($\gamma =3.1$^23^, $\gamma =3.9\pm 0.12$^24^) and other mammalian vasculatures where $\gamma$ can range as high as 4^6^. Such large $\gamma$ are not predicted by single segment analyses including effects related to pulsatile flow, elastic vessel walls and turbulence ($\gamma =2.3$)^6^. Tree complexity and organism imposed boundary conditions provide an additional contribution that can account for larger values of $\gamma _{\mathrm{opt}}$.

We predict that tree structures within the physiological regime, $2<\gamma <4$, are weakly dependent on all parameters except $\gamma$; outside the physiological regime structures also depend strongly on $\Omega$; and for all regimes tree structures are independent of *N*. Changes in *N* do not qualitatively change the morphology of the tree, but add more detail. Outside the regime $2<\gamma <4$, structure can change dramatically with $\Omega$.

For $2.5<\gamma <3.5$, we find length exponents in our computational trees that are consistent with the value $\alpha \sim 1$ obtained experimentally. Experimental values range from $0.85<\alpha <1.21$^6^. We find a similar range of values in our numerical calculations, and with improved description of the flow, the accuracy of the predictions could be improved. Values of $\alpha$ could be useful as input to other calculations.

Accurate values of $\gamma _{\mathrm{opt}}$ are particularly relevant to computational techniques used for growing very large arterial trees *in-silico*, such as constrained constructive optimization (CCO). Such algorithms rely upon a fixed bifurcation exponent to set the radii in the generated trees^19,25,26^. Similarly, allometric scaling arguments require knowledge of $\gamma$^7^, and variations of $\gamma _{\mathrm{opt}}$ could modify such approaches. $\gamma _{\mathrm{opt}}$ is quite hard to measure experimentally, and we consider the calculation of such values to be a useful application of our technique.

Future work to include additional physics, such as pulsatile flow, turbulence and vessel elasticity, would lead to a computational model with enhanced predictive power. These improvements to the treatment of flow through vessels could be incorporated into both the analytic expressions derived in this paper, and into the cost function of SALVO without having to change the core algorithm. Once analytical expressions are modified to include this additional physics, we suggest that parameters such as $m_{b}$ could be determined from empirical results. The significant structural changes visible at $\gamma \sim 2$ and $\gamma \sim 4$ would also be interesting areas for further study, since the rapid changes in the tree morphology are reminiscent of a phase transition. These changes are on the edge of the physiologically relevant regime. Confirmation of a phase transition would require the identification of an order parameter and the signatures of critical behaviour.

Finally, we hypothesise that evolutionary compromises may favour closer adherence to the predictions of single segment analyses in organs with large flow demands to the detriment of less flow-hungry organs. Additional studies could be carried out to test this hypothesis. Overall, the computational and analytical approaches introduced here lead to a range of predictions regarding the structures of vascular trees, that provide interesting links to experimental and theoretical approaches.

Fundamental biophysical understanding of complex vascular structure has applications to modelling of cardiovascular systems and diseases. Computational techniques and analytic expressions for describing complex and multiscale networks have potential applications in computer modelling of physiology^8,9,18^, medical imaging^18^ and diagnosis of cardiovascular disease^27^. Beyond the desire to understand the fundamental biological properties of vascular networks, deviations from optimal flow conditions could be a sign of underlying disease^27^. Another application of arterial growth algorithms is the correction of gaps in computed tomography and magnetic resonance angiography on small length scales^18^.

## Data Availability

The datasets generated and analysed during the current study are available in the ORDO repository: 10.21954/ou.rd.12220490.
